# Biological pathways related to mirna-125a-5p in behavioral variant of frontotemporal dementia

**DOI:** 10.1007/s11033-026-11965-x

**Published:** 2026-06-03

**Authors:** Jessica Diniz Pereira, Leticia Moreira Silva, Mhai Ozawa, Jéssica Abdo Gonçalves Tosatti, Stephanny Pinto Oliveira Lima, Isabela Resende Silva, Vinicius Ribeiro Jeunon, Maria das Graças Carvalho, Elisa de Paula França Resende, Leonardo Cruz de Souza, Paulo Caramelli, Karina Braga Gomes

**Affiliations:** 1 Faculdade de Farmácia, Universidade Federal de Minas Gerais, Belo Horizonte, Minas Gerais Brazil; 2 Faculdade de Medicina, Universidade Federal de Minas Gerais, Belo Horizonte, Minas Gerais Brazil; 3 Departamento de Análises Clínicas e Toxicológicas - Faculdade de Farmácia, Universidade Federal de Minas Gerais, Avenida Presidente Antônio Carlos, 6627 - Pampulha, Belo Horizonte, CEP: 31270-901 Minas Gerais Brasil

**Keywords:** Frontotemporal dementia behavioral variant, miRNAs, Biological pathways

## Abstract

**Background:**

Frontotemporal dementia (FTD) comprises a group of neurodegenerative disorders that lead to progressive changes in language, behavior, personality, and movement. Accounting for approximately 60% of cases, the behavioral variant (bvFTD) represents the most common clinical presentation of FTD. MicroRNAs (miRNAs) are small non-coding RNAs involved in gene regulation and are expressed in various biological fluids. Considering bvFTD as an inflammatory disorder, this study investigated the expression of inflammation-related miRNAs in individuals with bvFTD compared with age- and sex-matched cognitively healthy controls.

**Methods:**

Twenty patients with bvFTD and 20 cognitively healthy controls, matched for age and sex, were included in the study. Serum levels of miRNA-30c-5p and miRNA-125a-5p were analyzed.

**Results:**

miRNA-125a-5p expression was upregulated in individuals with bvFTD compared with controls (fold change = 2.02). No significant differences between groups were observed for miRNA-30c-5p. An inverse correlation was identified between miRNA-125a-5p expression and age within the bvFTD group. Pathway enrichment analysis revealed that miRNA-125a-5p was significantly involved in the regulation of genes associated with insulin receptor signaling, SUMOylation, SUMO E3 ligase–mediated protein modification, SUMOylation of chromatin organization proteins, estrogen-dependent gene expression, extracellular exosome pathways, neuron migration, microtubule dynamics, and nervous system development.

**Conclusions:**

These findings suggest that miRNA-125a-5p may play an important role in FTD-related biological pathways, underscoring its potential as a molecular marker in the pathophysiology of this disorder.

**Supplementary Information:**

The online version contains supplementary material available at 10.1007/s11033-026-11965-x.

## Introduction

Neurodegenerative diseases are neurological conditions characterized by progressive and irreversible neuronal loss, which leads to cognitive, functional, and motor impairments [1]. Frontotemporal dementia (FTD) comprises a heterogeneous group of neurodegenerative disorders primarily affecting the frontal and temporal lobes, leading to progressive impairments in language, behavior, personality, and motor function [2]. FTD is the second most common form of early-onset dementia, affecting individuals under 65 years of age, and accounts for approximately 1.7% to 7% of all dementia cases [3, 4]. The behavioral variant of FTD (bvFTD) represents the most prevalent clinical subtype, comprising approximately 60% of cases. Its core clinical features include behavioral disturbances and executive dysfunction, such as apathy, altered eating behaviors, disinhibition, stereotyped actions, and reduced empathy [3, 4].

Although the etiology of FTD remains incompletely understood, a key neuropathological hallmark is frontotemporal lobar degeneration, which is classified based on the predominant protein aggregates present in neuronal inclusions, including TAR DNA-binding protein 43 (TDP-43), tau protein, and FET family proteins [5]. In addition, a substantial proportion of cases—approximately 40%—are familial, reflecting a strong genetic component [2]. Pathogenic variants in genes such as *MAPT*, *GRN*, and *C9orf72* have been implicated in FTD and are associated with abnormal accumulation of tau or TDP-43 proteins, thereby contributing to disease pathophysiology [2]. The definitive diagnosis of FTD requires the presence of a compatible clinical syndrome supported by genetic or neuropathological confirmation and is classified as possible, probable, or definite according to established criteria [6].

Various studies have sought the early diagnosis of neurodegenerative diseases using different molecular approaches. Among them, the analysis of microRNAs (miRNAs), such as miR-125b-5p and miR-146a-5p, whose alterations in biological fluids have been associated with dementia [7, 8]. More recently, the role of mitochondrial microRNAs (mitomiRs) has also been investigated, expanding the understanding of the cellular mechanisms involved and their potential as early biomarkers and therapeutic targets [9].

MicroRNAs (miRNAs) are small non-coding RNAs that play a key role in post-transcriptional gene regulation and have emerged as promising candidates for both diagnostic biomarkers and mechanistic insights in neurodegenerative diseases [10]. Several studies have explored the potential of circulating miRNAs in FTD. For example, miRNA-320a and miRNA-92a-3p have been reported to be upregulated in plasma-derived extracellular vesicles from patients with FTD [11, 12]. Conversely, circulating miRNAs such as miR-127-3p, miR-663a, miR-502-3p, and miR-206 have been described as downregulated in individuals with FTD compared to healthy controls [13, 14].

From a biological and clinical perspective, the bvFTD subtype exhibits relatively greater phenotypic and neuroanatomical homogeneity compared to other FTD variants. This characteristic enhances the interpretability of molecular findings, particularly when investigating circulating biomarkers such as miRNAs, which may reflect specific patterns of neurodegeneration. Therefore, restricting the analysis to bvFTD improves internal validity and enables a more precise characterization of disease-associated molecular alterations. Accordingly, the present study aimed to investigate the expression of miRNA-125a-5p and miRNA-30c-5p (both implicated in inflammatory pathways) in individuals with bvFTD compared to cognitively healthy controls. To our knowledge, this is the first study to examine these miRNAs in the context of bvFTD and to explore the biological pathways associated with their predicted target genes in this disease.

## Materials and methods

### Patients and samples

Twenty patients with bvFTD and twenty cognitively healthy individuals were included in this case-control study, matched for age and sex. The bvFTD individuals were recruited from the Behavioral and Cognitive Neurology Outpatient Clinic of the Hospital das Clínicas of the Universidade Federal de Minas Gerais (UFMG), in Belo Horizonte, Brazil, from 2016 to 2024.

The study was approved by the Ethics Committee of UFMG (CAAE-09638212.8.0000.5149) and all participants (or their legal representatives) signed the informed consent form before being included in the project. This study was conducted in accordance with the ethical principles outlined in the Declaration of Helsinki.

The diagnosis of probable bvFTD was established according to international consensus criteria [15], supported by a cerebrospinal fluid biomarker profile not consistent with AD, and in the absence of genetic testing for known pathogenic variants. The control group consisted of community-dwelling individuals who did not report subjective memory complaints and scored less than 25 points on the Memory Assessment Clinics Questionnaire (MAC-Q). Furthermore, only participants exhibiting normal global cognitive functioning - defined as scoring above the education-adjusted cut-off points on the Mini-Mental State Examination (MMSE) [16] - were included in the control group [17, 18].

In both groups, exclusion criteria comprised the presence of autoimmune or hepatic diseases, acute inflammation and anti-inflammatory use, stroke, a history of acute myocardial infarction, renal failure or thromboembolic events and cancer diagnosis within the past five years. Peripheral blood samples were placed in serum tubes, processed, and stored at -80 °C.

### Selection and expression of miRNAs

The human miRNA-125a-5p and miRNA-30c-5p compose a PCR-array expression kit (Qiagen) with miRNAs related to inflammatory pathways. These miRNAs were found to be differentially expressed in our previous analysis, which evaluated outcomes associated to subchronic inflammatory diseases in Brazilian population [19]. Since there is robust evidence that FTD also presents inflammatory mechanisms [20, 24], we hypothesized that these miRNAs might also be involved in FTD.

MiRNA extraction – The NucleoSpin^®^ miRNA Plasma kit (Macherey-Nagel) was used for 300uL serum miRNA extraction according to the provided protocol. The miRNA samples were quantified using the NanoDrop Lite instrument (Thermo Scientific).

RT-PCR – Starting with 15 ng of extracted miRNA, cDNA synthesis was performed using the TaqMan^®^ Advanced miRNA cDNA Synthesis kit (Thermo Scientific). For the PCR reactions, the TaqMan^®^ Advanced miRNA assay line (20x) was used for miRNA-484; miRNA-125a-5p; and miRNA-30c-5p. The manufacturer’s instructions were followed. The 2^-ΔΔCt method was used for relative expression analysis, and the results were normalized using the endogenous control miRNA-484. The spike-in cel-miR-39 was added in extraction and amplification steps, as an internal control for normalization.

Efficiency – cDNA from a representative sample was used for primer efficiency calculations. The sample was diluted 10 times in series, and then a standard curve was generated for each primer used in the experiments. All samples were subjected to the same PCR conditions, as described previously. The formula used for the calculation was E = [(10^−1/slope − 1) × 100] [21]. The assays demonstrated efficiencies between 90% and 110% (Supplementary material 1).

### Enrichment analysis

Enrichment analysis was performed on the differentially expressed miRNAs using miRPath-v. 4.0 as the analysis tool. TarBase v. 8.0 database was selected to ensure reliable data with strong experimental validation. In TarBase targets, the Direct option was chosen, indicating that only experimentally confirmed interactions were considered. Regarding the analysis method, the Classic analysis was used, with a p-value threshold of 0.01 and false discovery rate (FDR) correction applied. Pathways were retrieved from the Gene Ontology (GO), KEGG, and Reactome databases. During the analysis, only pathways with FDR values ​​<0.05 were considered significant.

Data manipulation and figure construction were performed in R software, version 4.4.2, using tidyverse (v. 2.0.0) and readxl (v.1.4.5). Functional enrichment results were graphically represented using the ggplot2 (v. 4.0.2) package. Pathways and genes were constructed and visualized using the tidygraph (v.1.3.1) and ggraph (v. 2.2.1) packages (see https://github.com/jessidpereira/Script-for-the-construction-of-the-gene-and-pathway-network-based-on-miRNA-125a-5p..git).

### Statistical analysis

For statistical analysis, STATA software version 14.0 was used. The sample size was estimated from the mean values of ΔCt for the miRNA-30c-5p and miRNA-125a-5p, in agreement with the previous study [19]. The sample size calculation was performed using T test between two independent groups. The values considered were: power = 0.80; confidence interval = 0.95. The software used was OpenEpi. The ratio 1:1 case/control resulted in at minimum 20 individuals in each group.

Data normalization was verified using the Shapiro-Wilk test. Student’s t-test was used for parametric data and the Mann-Whitney test for non-parametric data. Fisher test was applied to categorical variables. Data expression was performed using mean ± standard deviation or median (interquartile range). Correlation data were verified using Spearman’s correlation. The effect size was calculated according to Cohen (1998) [22].

A multivariate logistic regression analysis was performed in two stages. First, a univariable model including miRNA-125a-5p ΔCt, MMSE and schooling were included in order to assess the independent association with bvFTD. The variables that presented *p* < 0.2 were included in the final linear regression model. This threshold is supported as a pragmatic approach to avoid premature exclusion of potentially relevant predictors and to better control for confounding effects. The model was considered appropriate when Homer & Lemeshow test (HLT) was > 0.05. A p-value < 0.05 was considered significant, while for gene expression, fold-change values ​​> 2 were considered the threshold for differential miRNA expression [23].

## Results

### Clinical characteristics of participants and miRNA expression

Table 1 summarizes the main characteristics of the study participants. The groups were matched for sex and age (*p* > 0.05). The control group exhibited a higher educational level compared to the bvFTD group (*p* = 0.037). As expected, MMSE scores were significantly lower in the bvFTD group than in controls (*p* = 0.008), and this difference remained significant after adjustment for educational level (data not shown).

**Table 1:** Characteristics of the participants – behavioral variant bvFTD and control groups

Variable	bvFTD (*n* = 20)	Control (*n* = 20)	*P* Value
Age (years)	64.4 ± 8.5	63.1 ± 10.8	0.680
Sex distribution - Male (N and %)	6 (30)	12 (60)	0.111
Female (N and %)	14 (70)	8 (40)	
Schooling			0.037*
1–4 years (%)	35^a^	5	
5–8 years (%)	5	0	
9–11 years (%)	10	35 ^a^	
>11 years (%)	50	60	
MMSE	25.0 (8.0)	29.5 (2.0)	0.008*

Parametric data are represented by mean ± standard deviation or median (interquartile range) and percentage for categorical variables. a = most frequent (adjusted test residual). MMSE: Mini-Mental State Examination.* p-value < 0.05 was considered significant

**Fig. 1:**
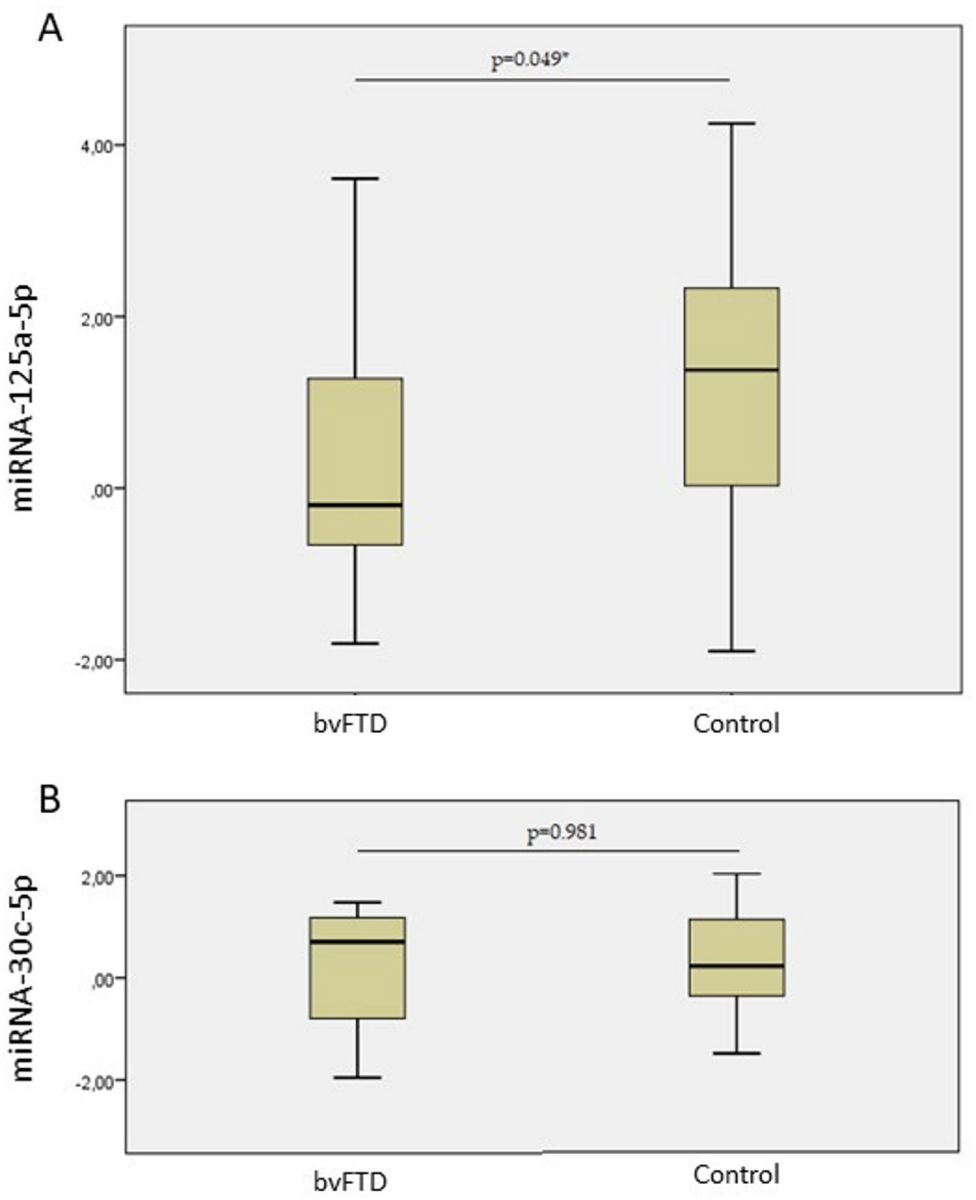
Serum relative quantification (ΔCt) of miRNA expression in patients with bvFTD compared to control subjects. (**A**) Relative expression of miRNA-125a-5p; (**B**) Relative expression of miRNA-30c-5p

miRNA-125a-5p was differentially expressed between groups, showing upregulation in the bvFTD group compared to controls (fold change = 2.02, *p* = 0.049). The effect size was moderate (*r* = 0.31) [24]. In contrast, no significant difference was observed for miRNA-30c-5p expression (fold change = 1.1, *p* = 0.981) (Fig. 1).

**Fig. 2:**
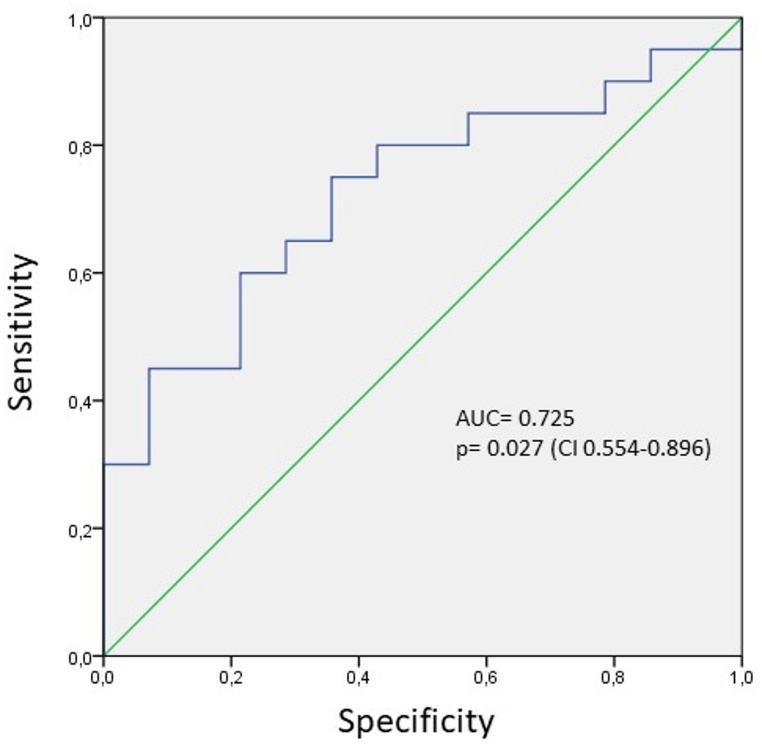
Receiver operating characteristic (ROC) curve analysis of miRNA-125a-5p for discriminating individuals with bvFTD from control subjects

Receiver operating characteristic (ROC) curve analysis evaluating the discriminative performance of miRNA-125a-5p yielded an area under the curve (AUC) of 0.725 (*p* = 0.027, 95% confidence interval: 0.554–0.896) (Fig. 2).

The association between miRNA-125a-5p expression and bvFTD remained statistically significant after adjustment for potential confounders, including MMSE score and educational level, in multivariable logistic regression analysis (ΔCt: *p* = 0.040; Hosmer–Lemeshow test *p* = 0.775). However, statistical significance was not retained after Bonferroni correction (adjusted significance threshold *p* < 0.02), indicating that this finding should be interpreted with caution and validated in larger cohorts (Supplementary Material 2).

**Fig. 3:**
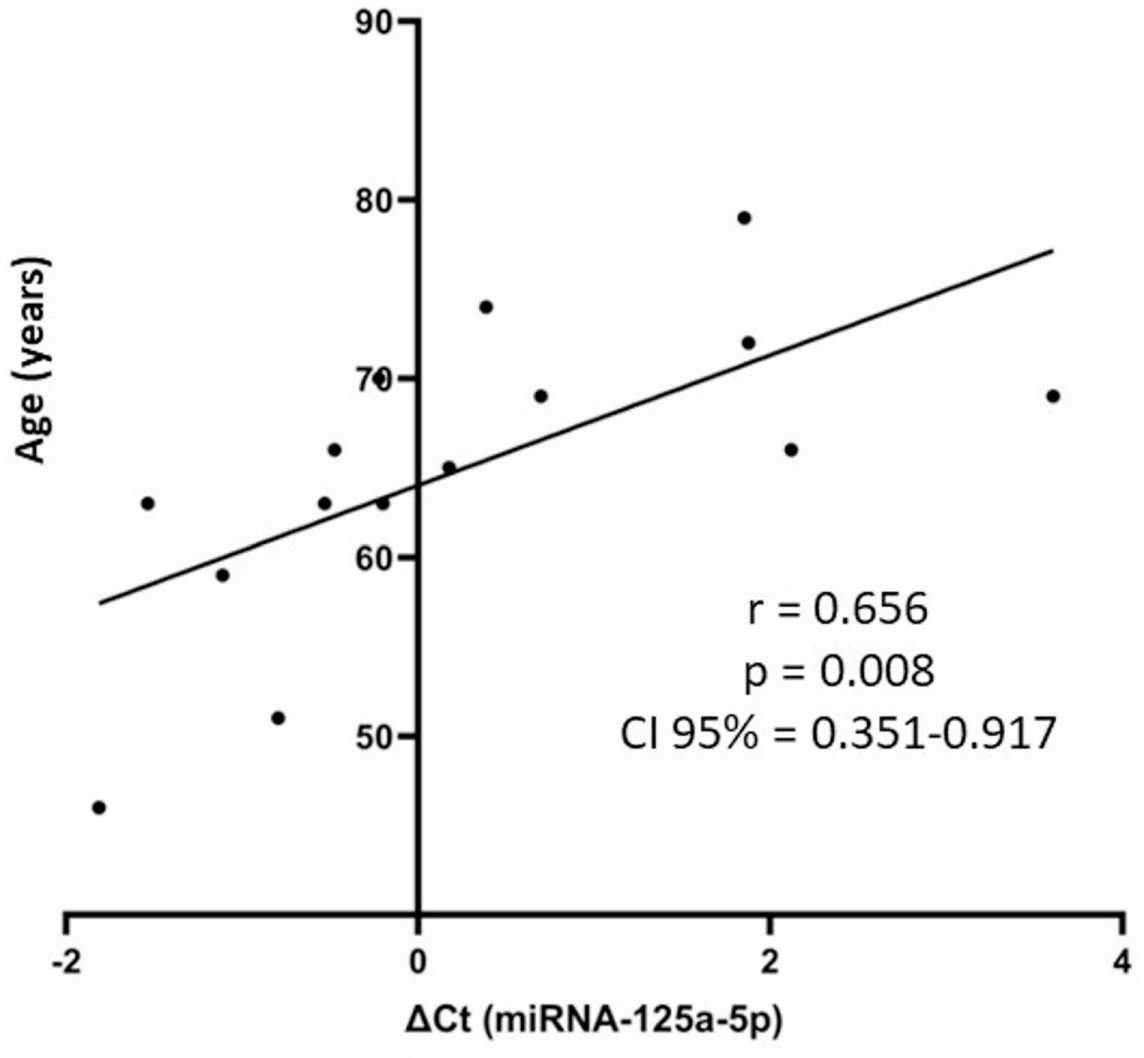
Correlation between miRNA-125a-5p ΔCt values and age in individuals with bvFTD

Interestingly, a significant positive correlation was observed between ΔCt values of miRNA-125a-5p and age in the bvFTD group (*r* = 0.656; *p* = 0.008), indicating an age-associated decrease in miRNA expression (Fig. 3). No such association was observed in the control group (*r* = 0.134; *p* = 0.574). No significant correlations were identified for miRNA-30c-5p.

### Pathway enrichment analysis

Pathway enrichment analysis was performed exclusively for miRNA-125a-5p, the only miRNA showing differential expression between groups. Broad and non-specific pathways, including those related to cancer and infectious diseases, were excluded to enhance biological interpretability. Importantly, these findings reflect predicted target gene enrichment derived from in silico analyses and should not be interpreted as evidence of direct mechanistic involvement.

**Fig. 4:**
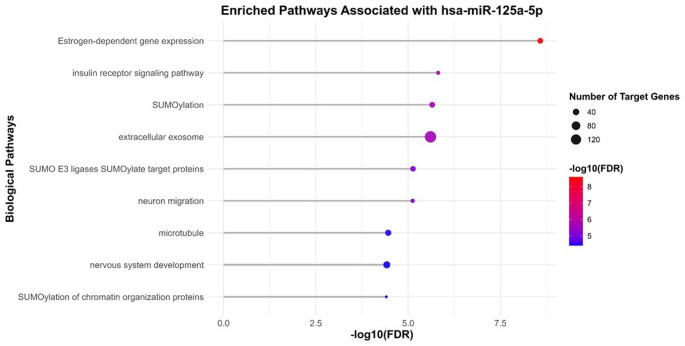
Enrichment analysis of the main biological pathways associated with miRNA-125a-5p in bvFTD. The lollipop plot illustrates the enrichment of biological pathways associated with predicted target genes of miRNA-125a-5p. Larger circles indicate stronger associations between target genes and their respective pathways. Pathways are ordered according to the number of associated target genes. The vertical axis represents the enrichment significance, expressed as −log10(FDR), with higher values indicating greater statistical significance (lower FDR)

**Fig. 5:**
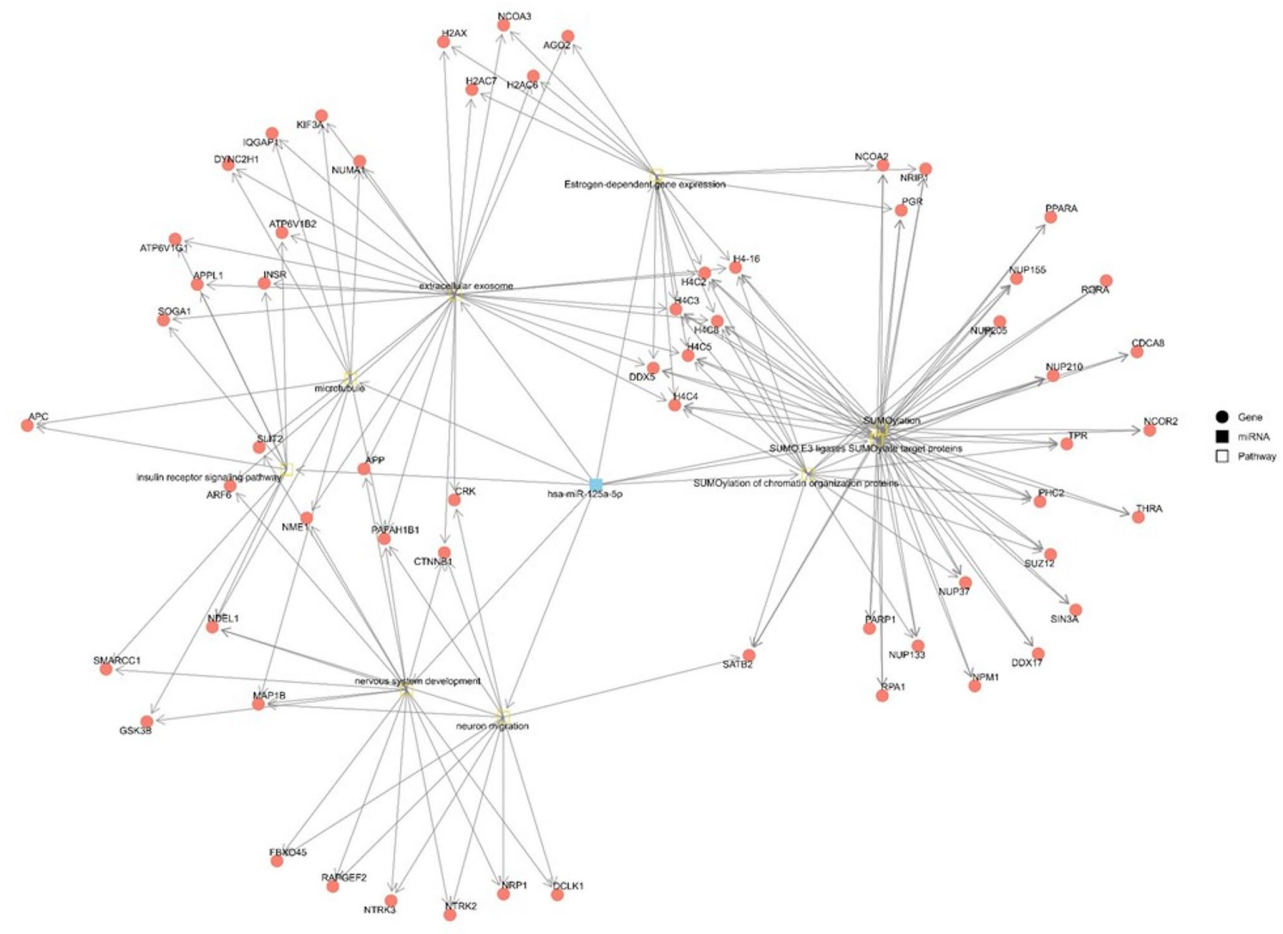
Network of interactions between miRNA-125a-5p, enriched signaling pathways, and shared target genes in bvFTD. The blue square represents miRNA-125a-5p, gold squares represent signaling pathways, and salmon-colored circles represent target genes shared across one or more pathways

Pathways with a merged false discovery rate (FDR) < 9 × 10⁻⁵ are presented in Supplementary Material 3. From these, the most biologically relevant pathways were selected along with their associated target genes (Figs. 4 and 5).

The main enriched pathways with potential relevance to bvFTD included insulin receptor signaling, SUMOylation-related processes (including SUMO E3 ligase activity and SUMOylation of chromatin organization proteins), estrogen-dependent gene expression, extracellular exosome pathways, neuron migration, microtubule organization, and nervous system development.

## Discussion

We investigated two microRNAs and their biological pathways in patients with bvFTD compared with cognitively healthy subjects. We observed that miRNA-125a-5p was upregulated in bvFTD and showed lower expression with age in this group. The relationship between miRNA-125a-5p and bvFTD provides new insights into possible genes related to the development of this disorder through biological pathways regulated by this miRNA.

The miRNA-125 family includes miRNAs 125a and 125b. miRNA-125a is located on chromosome 19 and is derived from the 5′ or 3′ arms, giving rise to two different mature miRNAs [24]. It has been observed that miR-125a-5p is involved in the function and development of immune cells and may exhibit tumor-suppressive or oncogenic functions [25].

The role of miRNA-125a-5p has been observed in several neurodegenerative diseases. In Parkinson’s disease (PD), miRNA-125a-5p was overrepresented in the plasma of patients compared to healthy controls [26]. It has been proposed that in cerebrospinal fluid (CSF), miRNA-125a-5p could be one of the miRNAs included in a genetic panel to differentiate healthy individuals from patients with Parkinson’s disease [27]. In individuals with Alzheimer’s disease (AD), plasma-derived extracellular vesicles showed increased levels of miRNA-125a-5p compared to cognitively healthy individuals [28]. However, another study reported that miRNA-125a-5p was downregulated in the serum of individuals with AD compared with cognitively healthy individuals [29]. In addition, in silico analyses indicated that miRNA-125a-5p targets the *PSEN2* and *APP* genes, both of which are associated with early-onset AD [30]. The study conducted by Derk et al. (2018) analyzed 10 different miRNAs in CSF of patients with FTD, AD and cognitively healthy individuals. It was observed that miRNA-125a-5p best discriminated between the FTD and control groups, with a sensitivity of 72% and specificity of 81% [31].

Our exploratory in silico approach reflects the signaling pathways anticipated to be involved in target regulation. This analysis showed that the main pathway identified and regulated by the miRNA-125a-5p target gene is the estrogen pathway. The relationship between estrogen and FTD is scarcely reported in the literature, however, interestingly, loss of progranulin (PGRN) gene, which is important for FTD, may be involved in preventing bone loss in female mice induced by aging and estrogen deficiency, an effect not observed in male mice [32]. Furthermore, PGRN has been shown to be a gene involved in sex steroids regulation in rats and to participate in brain masculinization during the perinatal period. In adult animals, gene expression has been reported to be positively regulated by estrogen in the hippocampus, suggesting that PGRN may be involved in mediating the mitogenic action of estrogen in neurogenic regions [33]. Regarding cognitive impairment, which is commonly observed in FTD, estrogen hormone therapy has been linked to an increased risk of cognitive impairment in women aged 65 years and older [34]. It has recently been described that older women over 70 years of age who underwent estrogen replacement therapy showed faster accumulation of tau protein compared with those who did not undergo therapy, mainly in the inferior temporal gyrus, fusiform gyrus, and entorhinal cortex [35].

Another pathway identified in the pathway enrichment analysis was insulin receptor signaling. Metabolic alterations have been reported in FTD, and it has been suggested that the insulin receptor may play a role in this condition. In tissues, higher expression of the insulin receptor (INSR) has been observed in individuals with FTD compared with controls [36]. There is also evidence of glucose hypometabolism in the frontal and temporal lobes, as well as increased expression of insulin and its receptors in tissues from patients with FTD [37, 38]. In blood samples, higher insulin levels and insulin resistance have been observed in patients with FTD compared with healthy controls [39]. These data suggest that dysregulation of insulin–IGF signaling networks may be related to cerebral hypometabolism and the neuropathological features of FTD [37].

MiRNA-125a-5p target genes are also involved in pathways where SUMO E3 ligases SUMOylate target proteins, SUMOylation and SUMOylation of chromatin organization proteins. SUMOylation is a reversible post-translational protein modification. It involves the binding of small ubiquitin-like modifier (SUMO) proteins to lysine residues of target proteins, where SUMO proteins are part of the ubiquitin-like protein (Ubls) family [40]. Five different SUMO paralogs were identified, including SUMO 2 and 3, which share 97% peptide sequence homology. The process occurs through the activation of an enzymatic cascade, which involves E1-activating enzymes, E3 SUMO ligases and E2-conjugating enzymes [40]. SUMOylation regulates important processes such as target protein trafficking and subcellular localization, solubility, stability, and structural conformations of protein activity and interactions [41]. In neurodegenerative diseases such as Parkinson’s, AD, Amyotrophic Lateral Sclerosis, and FTD, SUMOylation plays an important role [40, 42]. In FTD, Transactive Response DNA-binding protein 43 (TDP-43) is a protein that has a high propensity for aggregation due to its low solubility, and it can be altered as a result of the action of SUMOylation [43]. Interestingly, TDP-43 is also regulated by phosphorylation, ubiquitination, and acetylation, besides SUMOylation, and miRNA-125a-5p would be involved in the regulation of *HDAC4*,* HDAC5*, and *SMEK1*, thereby indirectly influencing post-translational modifications in cellular context. TDP-43 is essential for RNA binding; however, in disease states, it is found to be aggregated in the cytoplasm and displaced from the nucleus of neurons in the central nervous system (CNS) [42–44]. It is suggested that partial delocalization or complete depletion of TDP-43 is an early event in FTD and is strongly linked to protein aggregation, with TDP-43 being selectively modified by SUMO2 in response to cellular stressors [42]. SUMOylation by SUMO1 on a lysine residue would be responsible for its retention in the nucleus and, in this way, preventing aggregation in the cytoplasm [45]. Verde et al. (2025) reported that SUMO2/3 protein conjugation, in turn, would be responsible for a cellular mechanism capable of stabilizing the cytosolic RNA-free TDP-43 in response to cellular stress and, in this way, to avoiding protein aggregation [43]. Thus, understanding the regulatory mechanisms and functions of the pathways related to SUMOylation allows us to obtain important information about the disorder.

The extracellular exosome pathway was also identified during the analysis. It is seen that cells of the nervous system release exosomes, which influence gene expression and protein activities in recipient cells [46, 47]. In neurodegenerative diseases, it is observed that exosomes may be responsible for the induction of neuroinflammation and the transport of toxic and misfolded amyloid proteins [48]. In FTD, it is believed that the mechanisms of neurodegeneration could be caused by the depletion of PGRN in the brain [45]. This protein is generally secreted by the classical pathway and can also be released in association with exosomes [46]. Autosomal dominant mutations in GRN, the gene for PGRN, can trigger neural atrophy in the frontal and temporal lobes, which leads to FTD [49]. Benussi et al. (2016) reported that null mutations in GRN are strongly associated with changes in the composition and number of exosomes released, suggesting the role of exosomes in PGRN transportation [46]. Schneider et al. (2018) evaluated 15 symptomatic and 23 pre-symptomatic carriers of the FTD mutation, in addition to 11 healthy non-mutated carriers, using exosomes isolated from CSF. It was observed that the expression of the miRNAs miR-204-5p and miR-632 in exosomes was significantly reduced in symptomatic carriers of the mutation compared with pre-symptomatic carriers. To validate the findings, the expression among FTD, AD, and controls was also compared, and miRNA-623 showed decreased expression compared with the AD and control groups [50]. Another study in individuals with FTD showed that the expression of miRNA-320a in isolated exosomes from CSF was reduced in FTD patients compared with controls [51]. This suggests that exosomes in FTD play an important role in pathophysiology by carrying proteins or miRNAs related to the disease. However, serum miRNAs can originate from multiple peripheral sources, and future CSF/exosomal studies should be conducted in order to address this question.

PGRN is also involved in cell division, survival and neuron migration [50]. However, this does not apply to post-mitotic cells of the brain and spinal cord, many of which strongly express PGRN and are neither proliferative nor migratory [50]. In our study, one of the pathways identified was the neuron migration pathway. Therefore, further studies are needed to determine whether PGRN or other protein pathway are regulated by the miRNA-125a-5p.

The nervous system development pathway was also identified during the enrichment analysis. PGRN, in addition to being responsible for lysosomal biogenesis, inflammation, repair, stress response, and aging, also plays an important role in the development, survival, function, and maintenance of neurons [52]. Another important point in brain development is the phagocytic clearance of cellular dendrites and synapses by glia [54]. However, in FTD, it is observed that these processes are altered, which affects neural development and the maintenance of brain homeostasis [52].

Finally, the microtubule-related pathway was also identified. The microtubule-associated protein tau (MAPT) gene was the first gene identified with mutations that lead to FTD [53]. Mutations in tau can cause a decrease in the protein’s ability to bind to microtubules or a tendency toward increased aggregation and formation of hyperphosphorylated filaments [53]. Tau phosphorylation is one of the causes of decreased microtubule stabilization, as it disrupts the protein’s ability to bind to them and reduces tau solubility, making it more susceptible to aggregation [54]. Moreover, microtubule stability may also be involved in the regulation of miRNA-125a-5p.

The observed increase in miRNA-125a-5p expression (reflected by lower ΔCt values) in younger individuals exclusively within the bvFTD group suggests that this miRNA is more closely linked to disease-specific pathophysiological mechanisms than to chronological aging per se. This pattern is particularly noteworthy given that age is a major confounder in neurodegenerative disorders, yet the association here appears to be dissociated from typical age-related molecular changes. One plausible explanation is that its increased expression in younger bvFTD patients may reflect an early action preceding or paralleling overt neurodegeneration. Additionally, FTD is characterized by a relatively earlier age of onset compared with other dementias, such as AD, and often has a stronger genetic component. In this context, elevated miRNA-125a-5p levels in younger patients could indicate a more penetrant or biologically active disease rather than a simple age-dependent accumulation of molecular damage. Importantly, the absence of a similar association in controls strengthens the hypothesis that miRNA-125a-5p is not merely a phenomenon of aging, but rather a disease-linked molecular signal.

Taken together, these findings suggest that miRNA-125a-5p may participate in early, disease-specific molecular cascades in bvFTD, potentially related to neuroinflammation, genetic susceptibility, and dysregulated RNA metabolism. Its elevated expression in younger patients could reflect an early and more biologically active disease process, highlighting its potential utility as both a mechanistic marker and a candidate biomarker for early-onset bvFTD.

This study has several limitations, including the absence of a replication or validation cohort. The most relevant constraint is the limited number of patients and controls included in the analysis, as supported by the medium effect size observed. Additionally, there was a significant difference in educational level between groups, which may reflect socioeconomic or lifestyle factors associated with education and potentially influence miRNA expression. Furthermore, comprehensive miRNA profiling using next-generation sequencing or microarray approaches was not performed, precluding the assessment of the broader diagnostic potential of other miRNAs in bvFTD. Pathway analysis was restricted to a single miRNA, and only one normalizer was used. Moreover, bvFTD patients were not genetically characterized and miRNA changes may differ across genetic subtype, suggesting further investigation in diverse mutation carriers. miRNA expression was not normalized for the use of different medications, as this data is extremely variable. Finally, the biological sample included total circulating serum miRNAs, consequently, cerebrospinal fluid (CSF) specificity cannot be inferred and future CSF or neuron-derived exosome studies are needed. Therefore, the present findings should be interpreted as exploratory and warrant validation in independent studies with larger sample sizes.

Nonetheless, we anticipate that these exploratory results will stimulate further and more comprehensive investigations in this field. In addition, the therapeutic potential of miRNA-125a-5p to attenuate maladaptive inflammatory signaling, restore immune–neuronal communication, and modulate downstream pathways implicated in bvFTD progression requires extensive functional validation.

## Conclusions

miRNA-125a-5p is upregulated in bvFTD patients compared with controls and correlates inversely with age in the first group. Moreover, miRNA-125a-5p expression is related to the regulation of genes involved in the insulin receptor signaling pathway, SUMO E3 ligases SUMOylate target proteins, SUMOylation, SUMOylation of chromatin organization proteins, estrogen-dependent gene expression, extracellular exosome, neuron migration, microtubule and nervous system development pathways, which could be related to bvFTD pathophysiology.

This is the first study to demonstrate the involvement of miRNA-125a-5p in FTD, to the best of our knowledge, highlighting its potential relevance as a therapeutic target for the treatment of this disease.

## Electronic Supplementary Material

Below is the link to the electronic supplementary material.

Supplementary Material 1

## Data Availability

All data supporting the findings of this study are available within the paper and its Supplementary Information.

## Abbreviations

FTD: Frontotemporal dementia
AD: Alzheimer’s disease
bvFTD: Behavioral variant frontotemporal dementia
CSF: Cerebrospinal fluid
INSR: Insulin receptor
MAC-Q: Memory Assessment Clinics Questionnaire
MAPT: Microtubule-associated protein tau
miRNAS: MicroRNAs
MMSE: Mini-Mental State Examination
NGS: Next-generation sequencing
PD: Parkinson’s disease
PGRN: Progranulin
TDP-43: TAR DNA-binding protein 43
Ubls: Ubiquitin-like protein

## Code availability

https://github.com/jessidpereira/Script-for-the-construction-of-the-gene-and-pathway-network-based-on-miRNA-125a-5p..git.
