# Supplementary material for: Biological pathways related to mirna-125a-5p in behavioral variant of frontotemporal dementia
Source: Mol Biol Rep. 2026 Jun 3;53(1):877. doi: 10.1007/s11033-026-11965-x (PMC13234057; doi:10.1007/s11033-026-11965-x)
Supplement: Supplementary file 1 — Supplementary Material 1 [file 11033_2026_11965_MOESM1_ESM.docx]

**Supplementary material 1 -** Standard curves and efficiency (slope, R²) for each miRNA evaluated.


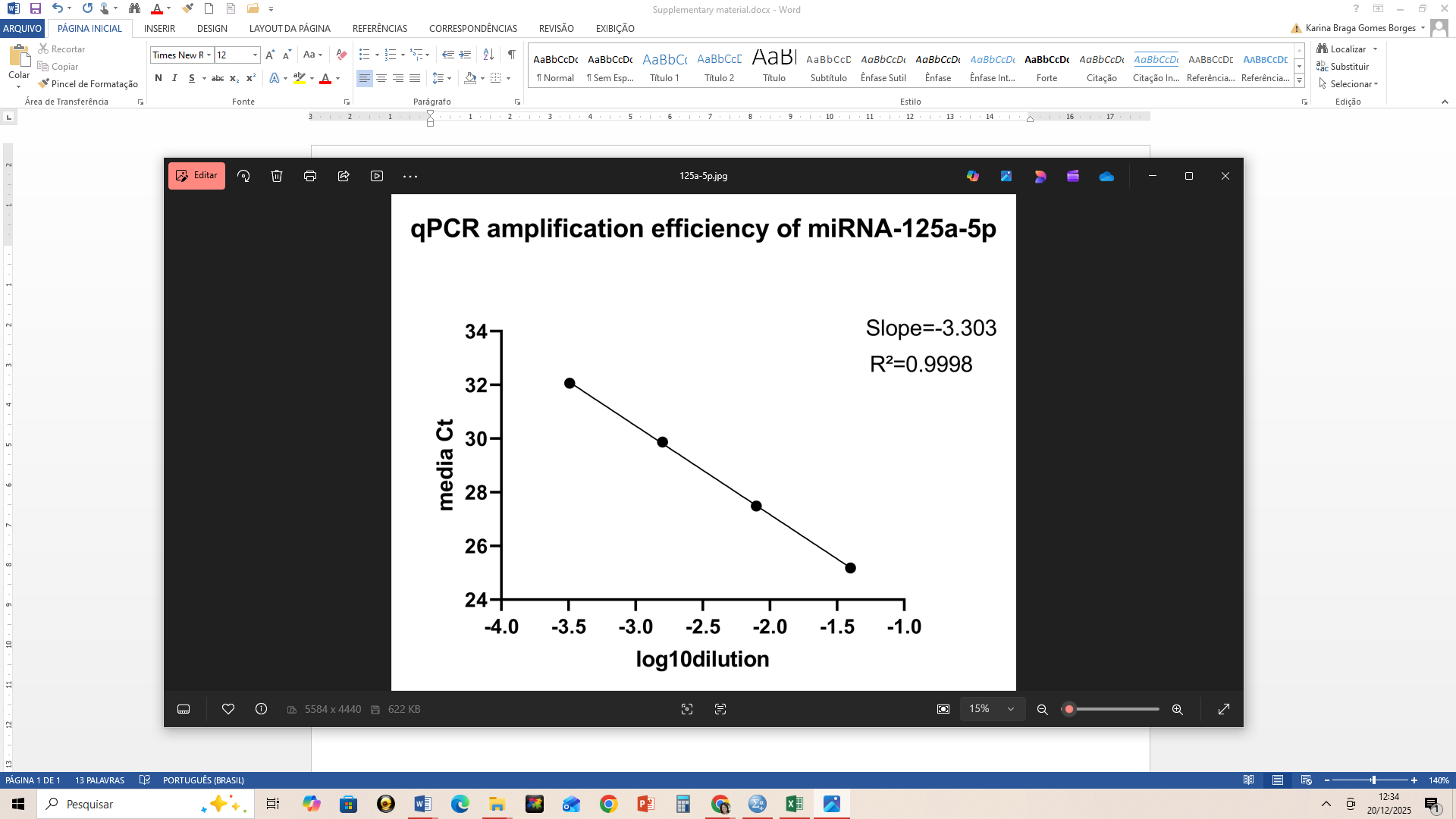


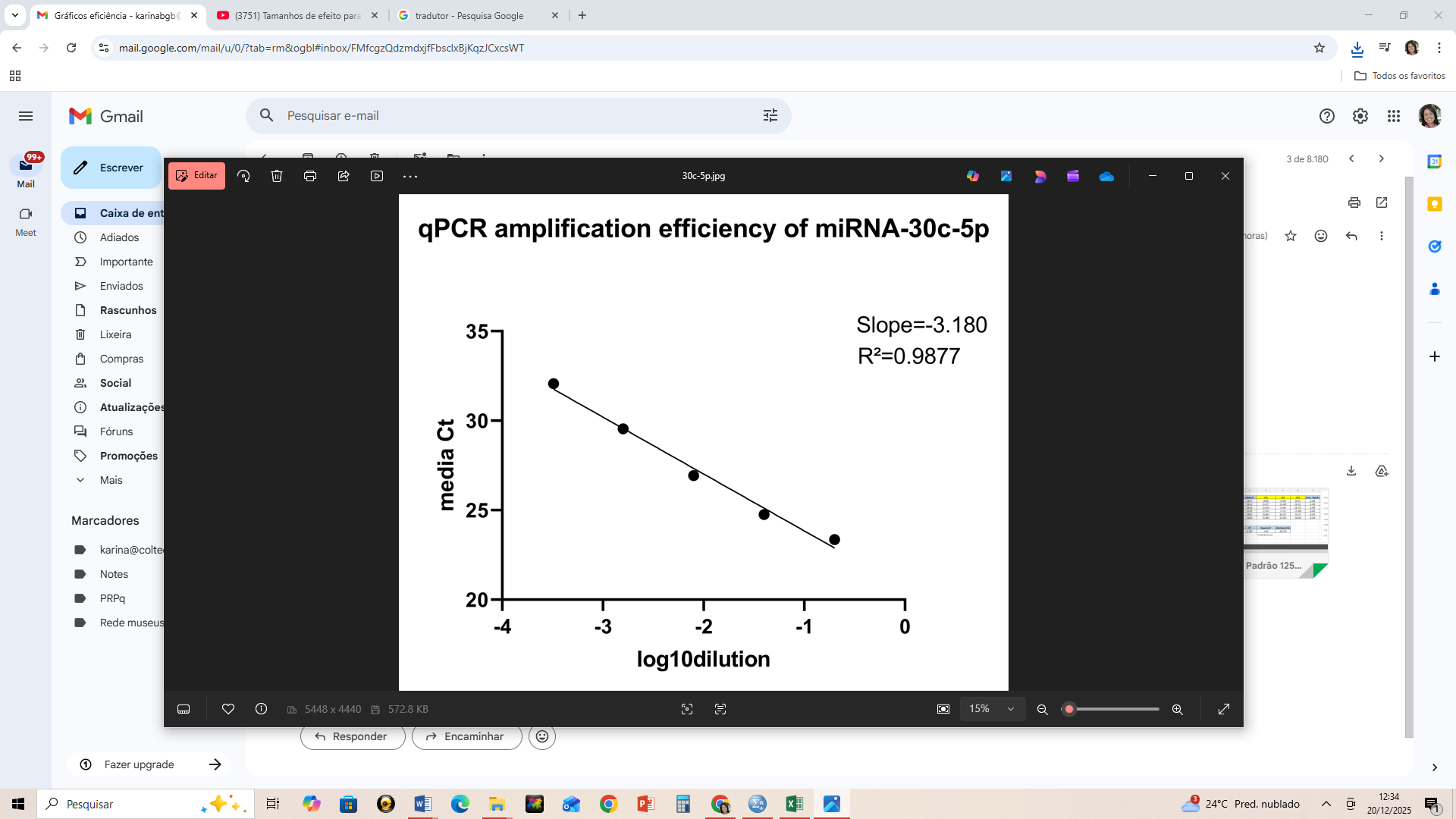


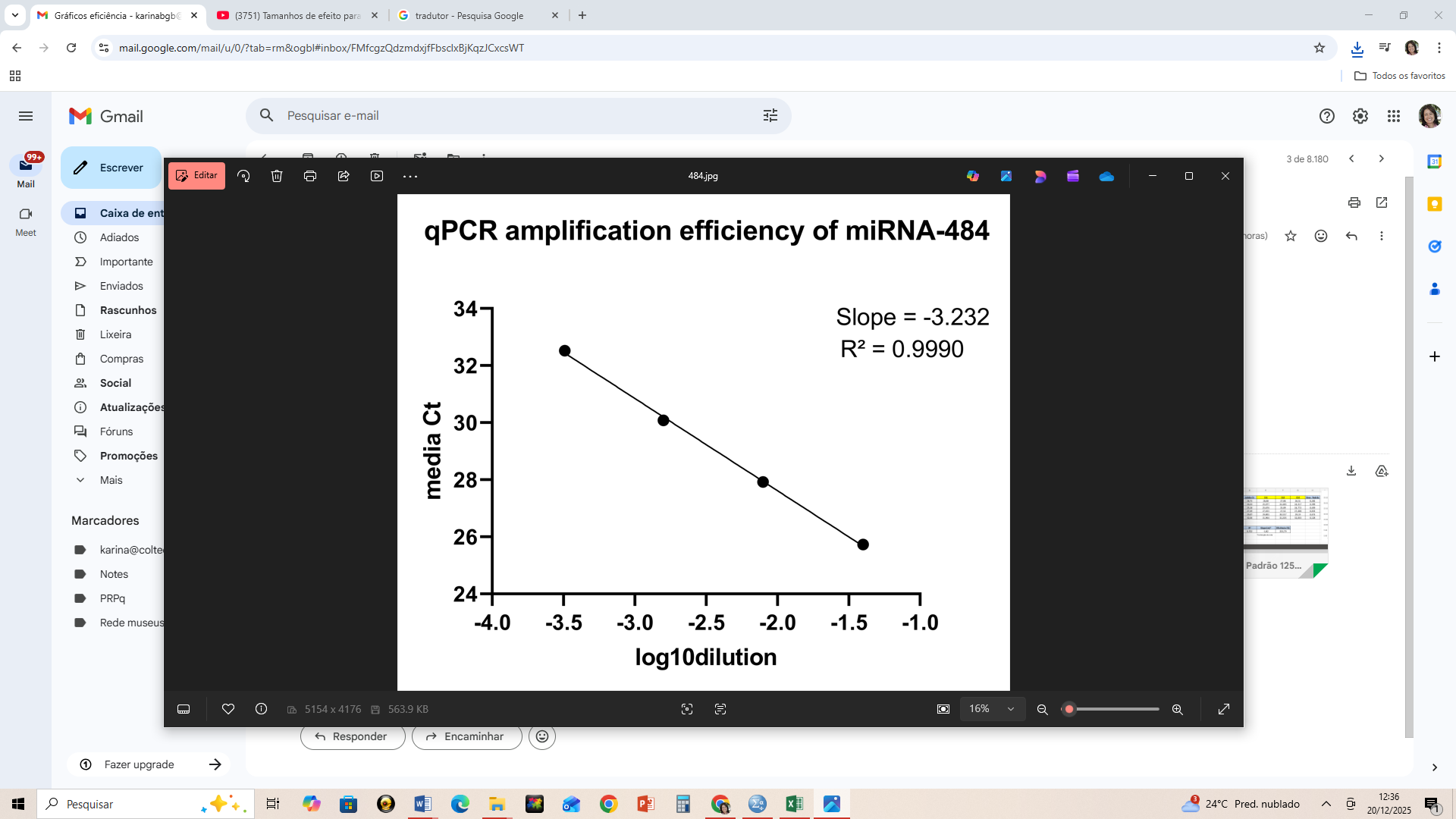


**Supplementary material 2 -** Multiple linear regression model for bvFTD and study variables.

| **Variable** |  | **OR** | **B** (unstandardized coeficiente) | **P** | **CI (95%)** |
| --- | --- | --- | --- | --- | --- |
| deltaCt miRNA-125a-5p |  | 4.617 | 1,53 | 0.040* | 1.075-19.836 |
| MMSE |  | 5.864 | 1,769 | 0.034* | 1.141-30.130 |
| Schooling (ages) |  | 0.059 | -2.825 | 0.214 | 0.001-2.547 |

MMSE - Mini-Mental State Examination; CI - confidence interval. * p significant <0.05, p significant after Bonferroni correction < 0.02.

**Supplementary material 3 -** Enrichment analysis.

| **Term Name** | **Term Genes** | **Target Genes (n)** | **P-value separate** | **Merged P-value** | **Merged FDR** | **Base** |
| --- | --- | --- | --- | --- | --- | --- |
| protein binding | 14297 | 831 | 1.19000530490684e-38 | 1,19001E-38 | 2,16581E-36 | GO |
| nucleus | 7177 | 483 | 1.30708285006485e-26 | 1,30708E-26 | 1,18945E-24 | GO |
| nucleoplasm | 4107 | 319 | 2.05048075092515e-25 | 2,05048E-25 | 1,24396E-23 | GO |
| cytosol | 5614 | 385 | 5.03151510085782e-21 | 5,03152E-21 | 2,28934E-19 | GO |
| RNA binding | 1707 | 162 | 3.55390678915764e-20 | 3,55391E-20 | 1,29362E-18 | GO |
| cytoplasm | 7391 | 469 | 9.83751676564057e-20 | 9,83752E-20 | 2,98405E-18 | GO |
| cadherin binding | 336 | 51 | 2.46657446944244e-14 | 2,46657E-14 | 6,41309E-13 | GO |
| positive regulation of transcription by RNA polymerase II | 1258 | 114 | 5.9644153814716e-13 | 5,96442E-13 | 1,3569E-11 | GO |
| Gene expression (Transcription) | 1661 | 157 | 1.65809594762104e-13 | 1,6581E-13 | 2,38766E-11 | REACTOME |
| RHO GTPase Effectors | 333 | 52 | 2.59743479046207e-12 | 2,59743E-12 | 1,87015E-10 | REACTOME |
| Generic Transcription Pathway | 1372 | 131 | 1.5425143560613e-11 | 1,54251E-11 | 7,40407E-10 | REACTOME |
| chromosome | 538 | 60 | 1.01117658789124e-10 | 1,01118E-10 | 2,04482E-09 | GO |
| ESR-mediated signaling | 226 | 39 | 7.41212152253836e-11 | 7,41212E-11 | 2,63165E-09 | REACTOME |
| Estrogen-dependent gene expression | 152 | 31 | 9.13766701698052e-11 | 9,13767E-11 | 2,63165E-09 | REACTOME |
| regulation of megakaryocyte differentiation | 71 | 19 | 2.09805474452519e-10 | 2,09805E-10 | 3,81846E-09 | GO |
| RNA Polymerase II Transcription | 1509 | 137 | 1.71499452869552e-10 | 1,71499E-10 | 4,11599E-09 | REACTOME |
| enzyme binding | 411 | 49 | 5.70690948057587e-10 | 5,70691E-10 | 9,44234E-09 | GO |
| perinuclear region of cytoplasm | 752 | 72 | 1.39186454744845e-09 | 1,39186E-09 | 2,11099E-08 | GO |
| Signaling by Rho GTPases | 476 | 59 | 1.28123659565428e-09 | 1,28124E-09 | 2,63569E-08 | REACTOME |
| chromatin organization | 402 | 47 | 2.43181948959399e-09 | 2,43182E-09 | 3,40455E-08 | GO |
| protein domain specific binding | 275 | 37 | 2.85170970288648e-09 | 2,85171E-09 | 3,70722E-08 | GO |
| protein-containing complex | 785 | 73 | 3.75183233907435e-09 | 3,75183E-09 | 4,55222E-08 | GO |
| DNA binding | 2466 | 171 | 4.59681194161177e-09 | 4,59681E-09 | 5,22887E-08 | GO |
| Signaling by Nuclear Receptors | 318 | 44 | 6.57228409168623e-09 | 6,57228E-09 | 1,18301E-07 | REACTOME |
| Transcriptional Regulation by TP53 | 427 | 53 | 8.85612147023607e-09 | 8,85612E-09 | 1,41698E-07 | REACTOME |
| double-stranded RNA binding | 72 | 17 | 1.48631154798321e-08 | 1,48631E-08 | 1,59123E-07 | GO |
| Chromatin modifying enzymes | 292 | 41 | 1.42537099373328e-08 | 1,42537E-08 | 1,86594E-07 | REACTOME |
| Chromatin organization | 292 | 41 | 1.42537099373328e-08 | 1,42537E-08 | 1,86594E-07 | REACTOME |
| RNA polymerase II cis-regulatory region sequence-specific DNA binding | 694 | 64 | 4.81900168686672e-08 | 4,819E-08 | 4,87255E-07 | GO |
| RUNX1 regulates genes involved in megakaryocyte differentiation and platelet function | 100 | 21 | 5.89861001327132e-08 | 5,89861E-08 | 6,70076E-07 | REACTOME |
| Signaling by Receptor Tyrosine Kinases | 528 | 59 | 6.04930169247922e-08 | 6,0493E-08 | 6,70076E-07 | REACTOME |
| viral process | 658 | 61 | 8.29428643918216e-08 | 8,29429E-08 | 7,56534E-07 | GO |
| positive regulation of axon extension | 39 | 12 | 8.31355609512771e-08 | 8,31356E-08 | 7,56534E-07 | GO |
| regulation of gene silencing by miRNA | 82 | 17 | 1.16135367990347e-07 | 1,16135E-07 | 1,00651E-06 | GO |
| protein C-terminus binding | 202 | 28 | 1.25673875583371e-07 | 1,25674E-07 | 1,03967E-06 | GO |
| protein transport | 714 | 64 | 1.36141322709787e-07 | 1,36141E-07 | 1,07729E-06 | GO |
| Cellular Senescence | 214 | 32 | 1.30636208828146e-07 | 1,30636E-07 | 1,34369E-06 | REACTOME |
| ribonucleoprotein complex | 182 | 26 | 1.92001844585484e-07 | 1,92002E-07 | 1,45601E-06 | GO |
| insulin receptor signaling pathway | 95 | 18 | 2.10336415302382e-07 | 2,10336E-07 | 1,53125E-06 | GO |
| regulation of transcription by RNA polymerase II | 918 | 76 | 2.34055442229506e-07 | 2,34055E-07 | 1,63839E-06 | GO |
| Cell Cycle | 764 | 75 | 2.1422432455278e-07 | 2,14224E-07 | 2,05655E-06 | REACTOME |
| SUMOylation | 209 | 31 | 2.47161060246274e-07 | 2,47161E-07 | 2,22445E-06 | REACTOME |
| Pre-NOTCH Expression and Processing | 128 | 23 | 2.85566024871077e-07 | 2,85566E-07 | 2,41891E-06 | REACTOME |
| extracellular exosome | 2384 | 158 | 3.6706318867191e-07 | 3,67063E-07 | 2,47428E-06 | GO |
| Cell Cycle Checkpoints | 326 | 41 | 3.15841999735196e-07 | 3,15842E-07 | 2,52674E-06 | REACTOME |
| protein-containing complex binding | 417 | 43 | 4.1763216902632e-07 | 4,17632E-07 | 2,71461E-06 | GO |
| positive regulation of cell migration | 267 | 32 | 4.89270645098661e-07 | 4,89271E-07 | 3,0299E-06 | GO |
| positive regulation of nuclear-transcribed mRNA poly(A) tail shortening | 13 | 7 | 4.99433675998529e-07 | 4,99434E-07 | 3,0299E-06 | GO |
| Gene Silencing by RNA | 141 | 24 | 4.48257162043935e-07 | 4,48257E-07 | 3,39732E-06 | REACTOME |
| Post-transcriptional silencing by small RNAs | 8 | 6 | 6.0350024053813e-07 | 6,035E-07 | 4,3452E-06 | REACTOME |
| negative regulation of transcription by RNA polymerase II | 896 | 73 | 7.45295811133879e-07 | 7,45296E-07 | 4,37561E-06 | GO |
| cell division | 428 | 43 | 8.43519465279112e-07 | 8,43519E-07 | 4,79752E-06 | GO |
| cytoskeleton | 1467 | 106 | 8.82012888518722e-07 | 8,82013E-07 | 4,86443E-06 | GO |
| nucleic acid binding | 1579 | 112 | 1.0362752480335e-06 | 1,03628E-06 | 5,54712E-06 | GO |
| Cell Cycle, Mitotic | 612 | 62 | 9.11899143194244e-07 | 9,11899E-07 | 6,25302E-06 | REACTOME |
| Mitotic Prophase | 147 | 24 | 9.82186150220969e-07 | 9,82186E-07 | 6,42885E-06 | REACTOME |
| RMTs methylate histone arginines | 81 | 17 | 1.08446364190933e-06 | 1,08446E-06 | 6,78969E-06 | REACTOME |
| chromatin | 152 | 22 | 1.31527039162557e-06 | 1,31527E-06 | 6,83941E-06 | GO |
| Transcriptional regulation by small RNAs | 109 | 20 | 1.20398948908435e-06 | 1,20399E-06 | 7,22394E-06 | REACTOME |
| Cellular responses to stress | 673 | 66 | 1.2637450046608e-06 | 1,26375E-06 | 7,27917E-06 | REACTOME |
| SUMO E3 ligases SUMOylate target proteins | 203 | 29 | 1.33176547485436e-06 | 1,33177E-06 | 7,37593E-06 | REACTOME |
| neuron migration | 119 | 19 | 1.53320134991104e-06 | 1,5332E-06 | 7,531E-06 | GO |
| chromatin binding | 513 | 48 | 1.54172345834147e-06 | 1,54172E-06 | 7,531E-06 | GO |
| focal adhesion | 468 | 45 | 1.57240560936891e-06 | 1,57241E-06 | 7,531E-06 | GO |
| mRNA binding | 229 | 28 | 1.67835062981063e-06 | 1,67835E-06 | 7,8323E-06 | GO |
| Competing endogenous RNAs (ceRNAs) regulate PTEN translation | 9 | 6 | 1.727924679151e-06 | 1,72792E-06 | 9,2156E-06 | REACTOME |
| Membrane Trafficking | 653 | 64 | 1.88989120955946e-06 | 1,88989E-06 | 9,71944E-06 | REACTOME |
| positive regulation of G2/M transition of mitotic cell cycle | 28 | 9 | 2.35084775245247e-06 | 2,35085E-06 | 1,06964E-05 | GO |
| kinetochore | 136 | 20 | 3.06746354495867e-06 | 3,06746E-06 | 1,26816E-05 | GO |
| early endosome | 304 | 33 | 3.01633956133271e-06 | 3,01634E-06 | 1,26816E-05 | GO |
| cell-cell junction | 210 | 26 | 3.09093770305367e-06 | 3,09094E-06 | 1,26816E-05 | GO |
| ruffle membrane | 102 | 17 | 2.94539673300926e-06 | 2,9454E-06 | 1,26816E-05 | GO |
| posttranscriptional gene silencing by RNA | 11 | 6 | 3.13556638114984e-06 | 3,13557E-06 | 1,26816E-05 | GO |
| Cellular responses to external stimuli | 687 | 66 | 2.58200807302187e-06 | 2,58201E-06 | 1,2821E-05 | REACTOME |
| cell cycle | 687 | 58 | 3.54131556558552e-06 | 3,54132E-06 | 1,35772E-05 | GO |
| neuron projection development | 149 | 21 | 3.50963302098666e-06 | 3,50963E-06 | 1,35772E-05 | GO |
| miRNA loading onto RISC involved in gene silencing by miRNA | 7 | 5 | 3.58081068320789e-06 | 3,58081E-06 | 1,35772E-05 | GO |
| nucleotide binding | 2030 | 134 | 4.00374380793747e-06 | 4,00374E-06 | 1,48596E-05 | GO |
| nucleolus | 939 | 73 | 4.08230812866614e-06 | 4,08231E-06 | 1,48596E-05 | GO |
| chromosome, centromeric region | 164 | 22 | 4.73693780054069e-06 | 4,73694E-06 | 1,68975E-05 | GO |
| RISC complex | 17 | 7 | 4.82786637754721e-06 | 4,82787E-06 | 1,68975E-05 | GO |
| chromatin silencing at rDNA | 38 | 10 | 4.92774048391001e-06 | 4,92774E-06 | 1,69217E-05 | GO |
| transcription coactivator activity | 258 | 29 | 5.99171416281112e-06 | 5,99171E-06 | 2,01943E-05 | GO |
| cytoplasmic stress granule | 76 | 14 | 6.57193088905068e-06 | 6,57193E-06 | 2,17471E-05 | GO |
| phosphorylation site mutants of CTNNB1 are not targeted to the proteasome by the destruction complex | 15 | 7 | 5.54869092469533e-06 | 5,54869E-06 | 2,28289E-05 | REACTOME |
| Misspliced GSK3beta mutants stabilize beta-catenin | 15 | 7 | 5.54869092469533e-06 | 5,54869E-06 | 2,28289E-05 | REACTOME |
| S33 mutants of beta-catenin aren't phosphorylated | 15 | 7 | 5.54869092469533e-06 | 5,54869E-06 | 2,28289E-05 | REACTOME |
| S37 mutants of beta-catenin aren't phosphorylated | 15 | 7 | 5.54869092469533e-06 | 5,54869E-06 | 2,28289E-05 | REACTOME |
| S45 mutants of beta-catenin aren't phosphorylated | 15 | 7 | 5.54869092469533e-06 | 5,54869E-06 | 2,28289E-05 | REACTOME |
| T41 mutants of beta-catenin aren't phosphorylated | 15 | 7 | 5.54869092469533e-06 | 5,54869E-06 | 2,28289E-05 | REACTOME |
| negative regulation of cell population proliferation | 480 | 44 | 7.05453424431102e-06 | 7,05453E-06 | 2,29272E-05 | GO |
| Epigenetic regulation of gene expression | 173 | 25 | 5.7746006715112e-06 | 5,7746E-06 | 2,30984E-05 | REACTOME |
| Post-translational protein modification | 1552 | 122 | 6.50437821671527e-06 | 6,50438E-06 | 2,53143E-05 | REACTOME |
| Regulation of actin cytoskeleton | 224 | 29 | 2.95618232257908e-06 | 2,95618E-06 | 2,66056E-05 | KEGG |
| negative regulation of gene expression | 334 | 34 | 8.77169975314054e-06 | 8,7717E-06 | 2,80079E-05 | GO |
| heart development | 251 | 28 | 9.94812561314321e-06 | 9,94813E-06 | 3,12165E-05 | GO |
| cell leading edge | 59 | 12 | 1.03527299890621e-05 | 1,03527E-05 | 3,13058E-05 | GO |
| positive regulation of transcription, DNA-templated | 663 | 55 | 1.04925957418322e-05 | 1,04926E-05 | 3,13058E-05 | GO |
| cellular response to nerve growth factor stimulus | 41 | 10 | 1.03335928720187e-05 | 1,03336E-05 | 3,13058E-05 | GO |
| Regulation of PTEN mRNA translation | 11 | 6 | 8.65762602094358e-06 | 8,65763E-06 | 3,28078E-05 | REACTOME |
| Oxidative Stress Induced Senescence | 134 | 21 | 8.99109895328668e-06 | 8,9911E-06 | 3,30764E-05 | REACTOME |
| Activation of anterior HOX genes in hindbrain development during early embryogenesis | 124 | 20 | 9.4175935416004e-06 | 9,41759E-06 | 3,30764E-05 | REACTOME |
| Activation of HOX genes during differentiation | 124 | 20 | 9.4175935416004e-06 | 9,41759E-06 | 3,30764E-05 | REACTOME |
| microtubule | 383 | 37 | 1.17812051832749e-05 | 1,17812E-05 | 3,45835E-05 | GO |
| PKMTs methylate histone lysines | 76 | 15 | 1.02861010222153e-05 | 1,02861E-05 | 3,52666E-05 | REACTOME |
| Transcriptional regulation by RUNX1 | 273 | 33 | 1.06011613645693e-05 | 1,06012E-05 | 3,55016E-05 | REACTOME |
| Mitotic Spindle Checkpoint | 115 | 19 | 1.09863940991158e-05 | 1,09864E-05 | 3,59555E-05 | REACTOME |
| nervous system development | 571 | 49 | 1.3067007611003e-05 | 1,3067E-05 | 3,77491E-05 | GO |
| Hippo signaling pathway | 164 | 23 | 8.39320451096205e-06 | 8,3932E-06 | 3,77694E-05 | KEGG |
| SUMOylation of chromatin organization proteins | 77 | 15 | 1.21510283668884e-05 | 1,2151E-05 | 3,88833E-05 | REACTOME |
| transcription factor binding | 401 | 38 | 1.39695398151024e-05 | 1,39695E-05 | 3,97259E-05 | GO |
| Signaling by NTRKs | 137 | 21 | 1.27742570242011e-05 | 1,27743E-05 | 3,9989E-05 | REACTOME |
| DNA-binding transcription activator activity, RNA polymerase II-specific | 479 | 43 | 1.49291489403606e-05 | 1,49291E-05 | 4,18016E-05 | GO |
| adherens junction | 177 | 22 | 1.63119463566807e-05 | 1,63119E-05 | 4,49814E-05 | GO |
| Beta-catenin phosphorylation cascade | 17 | 7 | 1.52528027834638e-05 | 1,52528E-05 | 4,6732E-05 | REACTOME |
| Amplification of signal from the kinetochores | 98 | 17 | 1.63970381675233e-05 | 1,6397E-05 | 4,81872E-05 | REACTOME |
| Amplification of signal from unattached kinetochores via a MAD2 inhibitory signal | 98 | 17 | 1.63970381675233e-05 | 1,6397E-05 | 4,81872E-05 | REACTOME |
| pre-miRNA processing | 14 | 6 | 1.81325227318003e-05 | 1,81325E-05 | 4,92555E-05 | GO |
| ATP binding | 1666 | 111 | 1.9737674344483e-05 | 1,97377E-05 | 5,10449E-05 | GO |
| Golgi apparatus | 1536 | 104 | 1.90833943866093e-05 | 1,90834E-05 | 5,10449E-05 | GO |
| corticospinal tract morphogenesis | 5 | 4 | 1.97588387535345e-05 | 1,97588E-05 | 5,10449E-05 | GO |
| RISC-loading complex | 9 | 5 | 1.99131081382548e-05 | 1,99131E-05 | 5,10449E-05 | GO |
| M Phase | 462 | 47 | 1.7958494123166e-05 | 1,79585E-05 | 5,17205E-05 | REACTOME |
| HCMV Late Events | 120 | 19 | 2.05358985213299e-05 | 2,05359E-05 | 5,79837E-05 | REACTOME |
| angiogenesis | 263 | 28 | 2.36609499258131e-05 | 2,36609E-05 | 5,89903E-05 | GO |
| dendrite | 504 | 44 | 2.36134018152401e-05 | 2,36134E-05 | 5,89903E-05 | GO |
| axon | 442 | 40 | 2.44277472785852e-05 | 2,44277E-05 | 6,00791E-05 | GO |
| gene silencing by RNA | 75 | 13 | 2.75738111035424e-05 | 2,75738E-05 | 6,69124E-05 | GO |
| apical part of cell | 97 | 15 | 2.80598710737279e-05 | 2,80599E-05 | 6,7196E-05 | GO |
| production of miRNAs involved in gene silencing by miRNA | 15 | 6 | 2.90678029876348e-05 | 2,90678E-05 | 6,87057E-05 | GO |
| axonal growth cone | 29 | 8 | 3.04405984565894e-05 | 3,04406E-05 | 7,0129E-05 | GO |
| mRNA stabilization | 29 | 8 | 3.04405984565894e-05 | 3,04406E-05 | 7,0129E-05 | GO |
| lamellipodium | 198 | 23 | 3.17393091561343e-05 | 3,17393E-05 | 7,22069E-05 | GO |
| RHO GTPases Activate Formins | 144 | 21 | 2.77194536517476e-05 | 2,77195E-05 | 7,67616E-05 | REACTOME |
| regulation of translation | 173 | 21 | 3.59973131183947e-05 | 3,59973E-05 | 8,08829E-05 | GO |
| beta-catenin binding | 88 | 14 | 3.72279947508937e-05 | 3,7228E-05 | 8,2628E-05 | GO |
| regulation of microtubule-based process | 10 | 5 | 3.83442251976605e-05 | 3,83442E-05 | 8,40801E-05 | GO |
| DNA Damage/Telomere Stress Induced Senescence | 83 | 15 | 3.10401015003051e-05 | 3,10401E-05 | 8,43354E-05 | REACTOME |
| multicellular organism development | 1220 | 85 | 4.24476435929669e-05 | 4,24476E-05 | 9,19699E-05 | GO |
| protein kinase binding | 501 | 43 | 4.34168325786482e-05 | 4,34168E-05 | 9,29631E-05 | GO |
| ATP-dependent microtubule motor activity, plus-end-directed | 16 | 6 | 4.47354773253885e-05 | 4,47355E-05 | 9,46728E-05 | GO |
| protein-DNA complex | 48 | 10 | 4.52715229175401e-05 | 4,52715E-05 | 9,47059E-05 | GO |
| rhythmic process | 150 | 19 | 4.6424393475756e-05 | 4,64244E-05 | 9,60141E-05 | GO |
| nuclear chromatin | 975 | 71 | 4.75156247408273e-05 | 4,75156E-05 | 9,71668E-05 | GO |
| glutamatergic synapse | 245 | 26 | 4.82034657224076e-05 | 4,82035E-05 | 9,74781E-05 | GO |
| positive regulation of cell population proliferation | 602 | 49 | 5.09425774930549e-05 | 5,09426E-05 | 0,000101885 | GO |
| nuclear chromosome | 59 | 11 | 5.64971347770786e-05 | 5,64971E-05 | 0,000110564 | GO |
| translation initiation factor activity | 59 | 11 | 5.64971347770786e-05 | 5,64971E-05 | 0,000110564 | GO |
| CRD-mediated mRNA stability complex | 6 | 4 | 5.71509850157745e-05 | 5,7151E-05 | 0,000110654 | GO |
| HCMV Infection | 171 | 23 | 4.37259614800147e-05 | 4,3726E-05 | 0,000116603 | REACTOME |
| AXIN mutants destabilize the destruction complex, activating WNT signaling | 14 | 6 | 4.89474262758595e-05 | 4,89474E-05 | 0,000117474 | REACTOME |
| truncated APC mutants destabilize the destruction complex | 14 | 6 | 4.89474262758595e-05 | 4,89474E-05 | 0,000117474 | REACTOME |
| AMER1 mutants destabilize the destruction complex | 14 | 6 | 4.89474262758595e-05 | 4,89474E-05 | 0,000117474 | REACTOME |
| APC truncation mutants have impaired AXIN binding | 14 | 6 | 4.89474262758595e-05 | 4,89474E-05 | 0,000117474 | REACTOME |
| AXIN missense mutants destabilize the destruction complex | 14 | 6 | 4.89474262758595e-05 | 4,89474E-05 | 0,000117474 | REACTOME |
| Truncations of AMER1 destabilize the destruction complex | 14 | 6 | 4.89474262758595e-05 | 4,89474E-05 | 0,000117474 | REACTOME |
| endosomal transport | 92 | 14 | 6.17064988927631e-05 | 6,17065E-05 | 0,000118217 | GO |
| chromatin silencing | 50 | 10 | 6.55220240531507e-05 | 6,5522E-05 | 0,000121597 | GO |
| endocytic recycling | 50 | 10 | 6.55220240531507e-05 | 6,5522E-05 | 0,000121597 | GO |
| platelet-derived growth factor receptor signaling pathway | 32 | 8 | 6.6143337991098e-05 | 6,61433E-05 | 0,000121597 | GO |
| positive regulation of intracellular protein transport | 24 | 7 | 6.51093795249309e-05 | 6,51094E-05 | 0,000121597 | GO |
| cell junction | 1004 | 72 | 6.81144029661258e-05 | 6,81144E-05 | 0,000122741 | GO |
| adherens junction assembly | 11 | 5 | 6.76847742114196e-05 | 6,76848E-05 | 0,000122741 | GO |
| positive regulation of translation | 82 | 13 | 7.26224380680884e-05 | 7,26224E-05 | 0,000128323 | GO |
| cellular response to growth factor stimulus | 71 | 12 | 7.20217866406842e-05 | 7,20218E-05 | 0,000128323 | GO |
| apoptotic process | 765 | 58 | 7.98508169343432e-05 | 7,98508E-05 | 0,000139739 | GO |
| positive regulation of neuron projection development | 131 | 17 | 8.44771465631891e-05 | 8,44771E-05 | 0,000146427 | GO |
| regulation of axonogenesis | 25 | 7 | 8.69266076949911e-05 | 8,69266E-05 | 0,000149251 | GO |
| protein import into nucleus | 95 | 14 | 8.83296490998125e-05 | 8,83296E-05 | 0,000150243 | GO |
| Resolution of Sister Chromatid Cohesion | 130 | 19 | 6.41005446825932e-05 | 6,41005E-05 | 0,000151319 | REACTOME |
| negative regulation of megakaryocyte differentiation | 18 | 6 | 9.59585708092654e-05 | 9,59586E-05 | 0,000161708 | GO |
| positive regulation of gene expression | 519 | 43 | 9.72198059686487e-05 | 9,72198E-05 | 0,00016233 | GO |
| P-body | 108 | 15 | 0.000100152109638199 | 0,000100152 | 0,000165706 | GO |
| polysome | 43 | 9 | 0.00010441703565914 | 0,000104417 | 0,000171206 | GO |
| Signaling by WNT | 366 | 38 | 7.41671201366896e-05 | 7,41671E-05 | 0,000172259 | REACTOME |
| Pre-NOTCH Transcription and Translation | 112 | 17 | 9.54164688645889e-05 | 9,54165E-05 | 0,000214687 | REACTOME |
| HCMV Early Events | 145 | 20 | 9.52691268477471e-05 | 9,52691E-05 | 0,000214687 | REACTOME |
